# A narrative review on the safety of glatiramer acetate in multiple sclerosis: focus on Europe

**DOI:** 10.1177/20406223251377965

**Published:** 2025-10-18

**Authors:** Refik Pul, Jelena Skuljec, Santosh B. Shirol, Riyaz A. Saboor, Christoph Kleinschnitz

**Affiliations:** Department of Neurology, Center for Translational Neuro- and Behavioral Sciences, University Medicine Essen, Hufelandstr. 55, Essen 45147, Germany; Department of Neurology, Center for Translational Neuro- and Behavioral Sciences, University Medicine Essen, Essen, Germany; Global NCD Excellence, Viatris, Bangalore, India; Global Product Safety and Risk Management, Viatris, Hyderabad, India; Department of Neurology, Center for Translational Neuro- and Behavioral Sciences, University Medicine Essen, Essen, Germany

**Keywords:** disease-modifying therapy, glatiramer acetate, immunomodulation, multiple sclerosis, non-biological complex drug, patient safety, relapsing/remitting, tolerability

## Abstract

Glatiramer acetate (GA) has been a pivotal therapy for relapsing multiple sclerosis (MS) due to its favorable safety profile. Long-term data spanning decades demonstrate its continued use in diverse patient populations. Adverse events include manageable localized injection site reactions, lipoatrophy or necrosis, and rare cases of liver injury. GA has minimal effects on immune function, and does not increase the risk of opportunistic infections, making it suitable for MS patients at risk for infections or reactivation of latent infections. GA’s immunomodulatory properties may pose a lower infection risk than other disease-modifying treatments. Progressive multifocal leukoencephalopathy risk with GA is low, and screening for latent infection is unnecessary before treatment. Vaccination is important for preventing infections in MS patients. GA does not compromise vaccine efficacy and is compatible with both inactivated and live attenuated vaccines. Special populations that may benefit from the characteristics of GA include older adults and patients with comorbidities and/or polypharmacy. MS patients often have comorbidities, necessitating careful management of potential drug interactions and side effects. Drug interactions with GA are not predicted, and clinical data suggest that the risk is low. GA is not contraindicated during pregnancy and exhibits a reassuring safety profile during breastfeeding, with no increased risk of adverse outcomes identified. Regulatory restrictions on GA use during breastfeeding have been removed. In summary, GA remains a safe and well-established therapy for MS patients, including those in special populations. Its favorable safety profile, compatibility with vaccination, and reassuring outcomes solidify its role in MS treatment.

## Plane language summary

Glatiramer acetate safety

Glatiramer acetate is a relatively safe DMT with minimal effects on immune function that does not compromise vaccine efficacy, making it suitable for MS patients at risk for infections or requiring vaccination. A reassuring safety profile and minimal drug interactions make it a viable treatment option for special populations common among MS patients, such as older adults and patients with comorbidities or polypharmacy. Use during pregnancy is not contraindicated, and should consider the benefit-risk assessment, while regulatory restrictions on its use in breastfeeding have been removed.

## Glatiramer acetate

Glatiramer acetate (GA) was shown to reduce relapse rate and MRI disease activity in controlled clinical trials,^1–4^ leading to its approval for reducing multiple sclerosis (MS) relapse rates and MRI disease activity in patients with relapsing MS in the United States in 1996,^5^ and in Europe in 2003.^6^

GA is a mixture of polypeptides with an average molecular weight of 5–9 kDa, comprising four naturally occurring amino acids. This non-biological complex drug (NBCD) is manufactured by the copolymerization of l-glycine, l-lysine, l-alanine, and l-tyrosine in a molar ratio of 1.9:4.6:6.0:1.0. Omission of any one amino acid resulted in loss of effect.^7^ In contrast to the original version, GA is currently produced with a molar ratio of 4.2:3.4:1.4:1, although the reason for this change remains unreported.^8^ The large number of possible components with different molecular weights and sequences precludes definitive structural elucidation by component analysis^9^; however, modern analytical and quantitative methods enabled reverse engineering of the synthesis procedure for the development of generics, ensuring equivalence with the originator. The development and production of follow-on GAs (FOGA) require a strictly controlled manufacturing process to avoid unanticipated consequences from even minor differences in the distribution of molecular weights or in the composition of antigenic polypeptide sequences, which can have an impact on the efficacy, toxicity, and immunogenicity profiles of the FOGA.^10^

**Figure 1.**
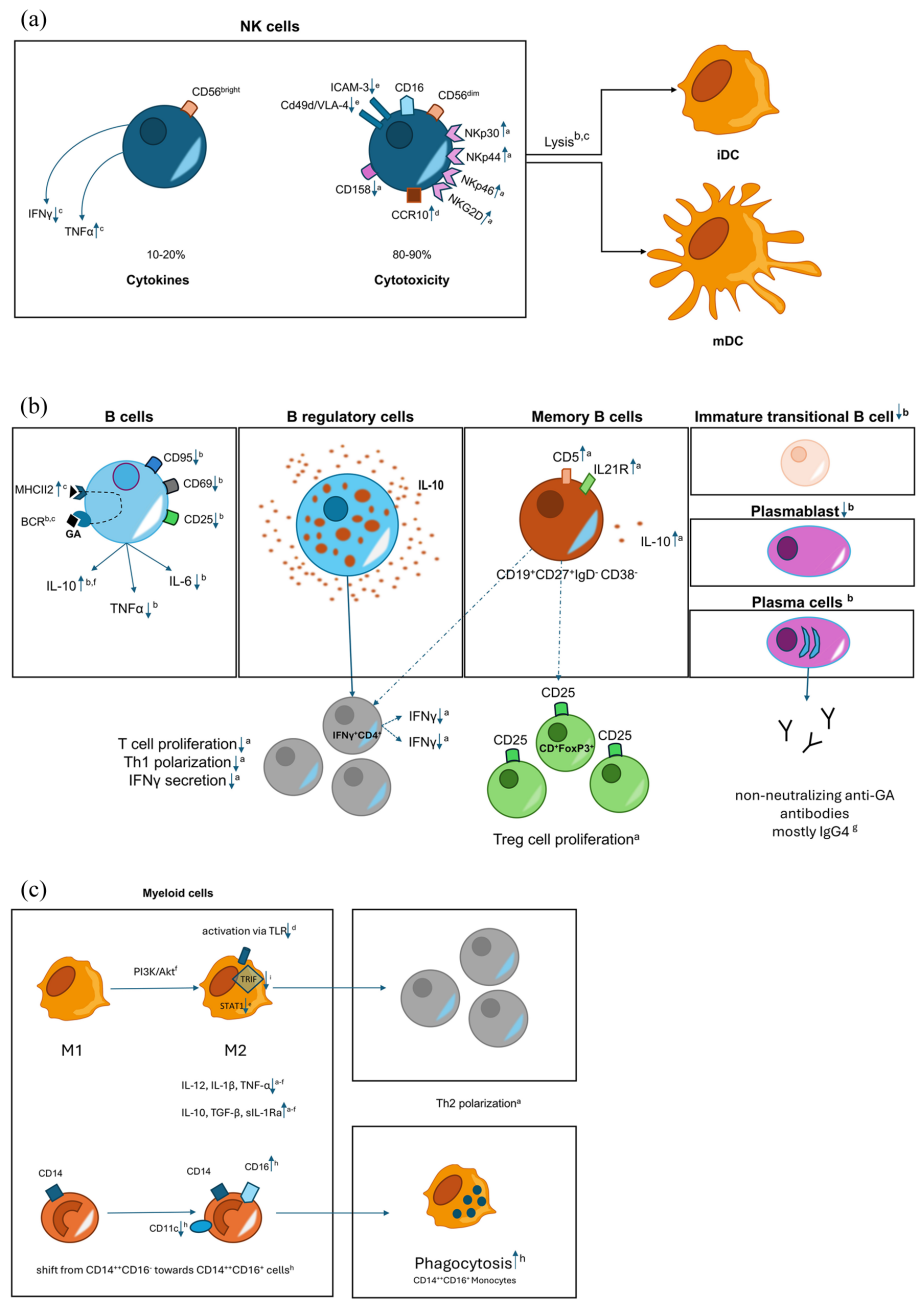
Effects of glatiramer acetate on immune cell populations. (a) Glatiramer acetate increases the biological activities of NK cells, particularly their cytolytic potential. ^a^Høglund et al.,^20^ ^b^Al-Falahi et al.,^19^ ^c^Sand et al.,^18^ ^d^Maghazachi et al.,^21^ and ^e^Sellner et al.^22^ (b) Glatiramer acetate increases anti-inflammatory and reduces proinflammatory cytokine levels. Glatiramer acetate-treated B memory and regulatory cells promote the formation of Th2 T cells and regulatory cells. ^a^Amrouche et al.,^23^ ^b^Häusler et al.,^24^ ^c^Jackson,^25^ ^d^Maghazachi et al.,^21^ ^e^Sellner et al.,^22^ ^f^Carrieri et al.,^26^ ^g^Sellebjerg et al.,^27^ and ^h^Harp et al.^28^ (c) Glatiramer acetate induces differentiation into type II (M2) myeloid cells and increases their phagocytic capacity. ^a^Vieira et al.,^29^ ^b^Jung et al.,^30^ ^c^Kim et al.,^31^ ^d^Weber et al.,^32^ ^e^Burger et al.,^33^ ^f^Carpintero et al.,^34^ ^g^Chabot et al.,^35^ ^h^Pul et al.,^15^ and ^i^Molnarfi et al.^36^ BCR, B cell receptor; ICAM-3, intercellular adhesion molecule-3; iDC, immature DC; IFNγ, interferon gamma; IL-10, interleukin-10; mDC, mature DC; TNFα, tumor necrosis factor alpha; Treg, regulatory T cell. Created with BioRender.com.

GA reduces inflammation by shifting reactive T cells toward a Th2 pattern, enhancing T regulatory cells (Tregs) and increasing anti-inflammatory cytokine production, while reducing the production of inflammatory cytokines.^11–14^ This Th2 skewing of the T cell response is one widely accepted mechanism of action for GA; however, a newer concept involves additional immunomodulatory effects on cells of the myelo-monocytic lineage through induction of anti-inflammatory “type II” antigen-presenting monocytes.^15,16^ In recent studies, GA treatment was linked to decreased number of plasmablasts, B cells, and memory B cells, in addition to a shift of B cells from a pro-inflammatory to an anti-inflammatory phenotype.^17^ GA enhances NK cell cytolysis of dendritic cells,^18–22^ perhaps by enhancing interaction through NK cytotoxicity receptors and reducing MHC class I expression on dendritic cells (Figure 1).

Possible neuroprotective effects of GA (seen mainly in animal models) may be associated with increased levels of neurotrophic factors secreted by Th2-biased immune cells. Effects of GA on the neuro-proinflammatory and degenerative processes implicated in MS-associated cognitive decline may help preserve nerve cell function and integrity.^37^ In experimental autoimmune encephalomyelitis (EAE), GA enhances the proliferation, migration, and differentiation of oligodendroglial and neuronal progenitor cells that foster repair, remyelination, and neurogenesis.^38,39^ GA protects neurons by increasing levels of neurotrophic factors and may reduce glutamate-mediated neurotoxicity.^40–42^ GA treatment reduced serum neurofilament light chain levels by 81% in EAE,^43^ suggesting that it may serve as a prognostic marker during GA treatment.

### Follow-on GAs

Access to disease-modifying therapies (DMTs) for MS is limited in many regions, especially in low- and middle-income countries,^44^ and stakeholders suggest that biosimilars/generics may improve affordability and access to DMTs for MS patients.^45–48^ Patent protection for Copaxone^®^ 20 mg daily (originator GA) ended in 2014. There has been debate on how to define and determine similarity between originator and FOGAs.^49–54^ The manufacturing process is fundamental to the production of NBCDs that maintain the characteristics of the originator product.^55^

Regulatory agencies have recognized several FOGAs as equivalent to the originator in terms of safety and efficacy. In April 2015, the US Food and Drug Administration (FDA) approved a generic form of GA (Glatopa™, originally produced by Momenta Pharmaceuticals), based on the synthesis and purification procedures used, and on its physicochemical, biological, and immunological properties in EAE.^56^ Subsequently, the FDA approved 20 and 40 mg formulations of a second generic GA originally produced by Natco Pharma in October 2017. These approvals were based on the Abbreviated New Drug Application process, which did not require additional clinical data.

In contrast, the European Medicine Agency (EMA) considered GA to be an NBCD and advised a third sponsor, Synthon, to demonstrate equivalent efficacy, safety, and tolerability of its generic GA product with new clinical data. These data were collected in the 9-month randomized, placebo-controlled, double-blind, Glatiramer Acetate Clinical Trial to Assess Equivalence With Copaxone (GATE) study,^57^ and 15-month extension.^58^ Patients aged 18–55 years old with relapsing MS (⩾1 relapse in the previous year and 1–15 gadolinium-enhancing MRI lesions) were randomly assigned to receive daily sc. injections of originator GA 20 mg (*n* = 357), FOGA 20 mg (*n* = 353), or placebo (*n* = 84). The study had an equivalence design and a primary endpoint of MRI-related outcome as a surrogate for relapses. Safety and tolerability were similar in both groups. Study discontinuation for any reason occurred in 3.4% of patients receiving FOGA and 1.1% receiving originator GA; however, discontinuation for any reason does not necessarily reflect a higher rate of adverse events, but could also result from a subjective tolerability issue. In the same study,^57^ the occurrence of any event was reported by 51.0% of subjects receiving the FOGA and 54.3% of subjects receiving the originator GA. Similarly, the percentages of subjects experiencing any serious adverse event (3.4% and 4.8%) and any severe event (4.0% and 2.8%) in the FOGA and originator GA, respectively, suggest that these variables are comparable between the two treatments.

**Figure 2.**
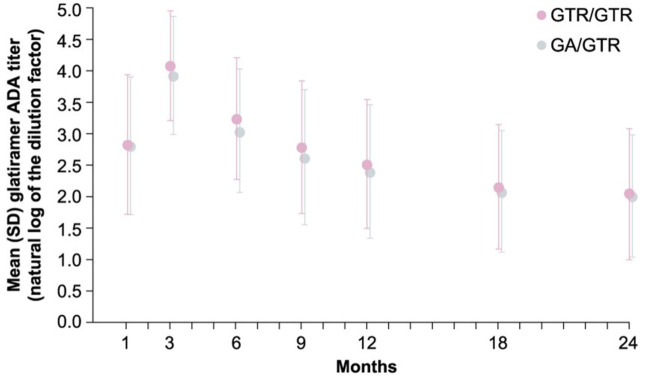
Glatiramer antibody (ADA) titer: mean (∓SD) ADA titer values in patients treated with generic glatiramer acetate (GTR) over 24 months and patients treated with brand glatiramer acetate (GA) for 9 months then switched to GTR for 15 months. Source: From figure 3 in *Mult Scler* 2017; 23(14): 1909–1917.58 Creative Commons Attribution-Non-Commercial 3.0 License http://www.creativecommons.org/licenses/by-nc/3.0/). Permission for this figure here. https://s100.copyright.com/AppDispatchServlet?publisherName=sageuk&publication=l979&title=Switching+from+branded+to+generic+glatiramer+acetate%3A+15-month+GATE+trial+extension+results&publicationDate=2017-12-01&author=Krzysztof+Selmaj%2C+Frederik+Barkhof%2C+Anna+N+Belova%2C+Christian+Wolf%2C+et+al.&contentID=10.1177%2F1352458516688956&volumeNum=23&issueNum=14&startPage=1909&endPage=1917&oa=cc-by-nc&orderBeanReset=true

All participants then received FOGA in the 15-month open-label extension,^58^ during which safety and tolerability, as well as the development of anti-drug antibodies, were found to be comparable in both incidence and titer (Figure 2).

Several studies using sophisticated methodologies have demonstrated differences in physico-chemical characteristics between originator GA and other glatiramoids.^59,60^ Studies have also reported different and sometimes contrasting effects on biological pathways relevant to the pathogenesis of MS (such as increased pro-inflammatory cytokine secretion by FOGAs) and effects on gene expression, raising concern about non-equivalent efficacy and antigenicity of FOGAs if not tested in appropriate clinical trials.^53,61–63^ In a genome-wide expression study performed in THP-1 cells by the company Teva (Teva Pharmaceutical Industries Ltd, Petach Tikva, Israel), originator GA modulated the expression of 6890 genes after 6 h of treatment, while treatment with the FOGA Probioglat^®^ (Probiomed, Mexico City, Mexico) resulted in differential expression of 136 genes at the same time point.^63^ The authors claim that Probioglat induced a more pro-inflammatory response compared to the originator GA. Similar shifts in expression of the genes identified (CCL5, CCL2, MMP9, MMP1, CXCL10, CD14, ICAM1, and BIRC3) were not observed in the study described above (CXCL10 was down-regulated by both originator and FOGA).^63^

A study by Hasson et al.^61^ showed differences between originator GA and the FOGA Polimunol^®^ (Synthon Bago, Autonomous City of Buenos Aires, Argentina). However, this study compared a single Polimunol batch vs. three Copaxone batches, and used a gene expression difference cut-off of 1.1-fold, which is not considered biologically relevant. Instead, the genome-wide expression study conducted by Kolitz et al. compared five FOGA batches with five originator GA batches, using 1.4-fold change cut-off value at a *p*-value of <0.05, which could be expected to provide more biologically meaningful results.^63^

Furthermore, Bakshi et al. conducted an investigation using splenocytes isolated from mice that had been immunized with the GA reference standard (Teva) and subsequently exposed *ex vivo* to various glatiramoids. Their comparison between GA-Teva and GA-Natco revealed significant differences in the expression of 98 genes (fold change ⩾ 1.3), which are primarily associated with T cell differentiation, immune suppression, antigen presentation, induction of Tregs, inflammatory responses, and cell adhesion.^62^ In a related study, Towfic et al. developed computational methods to assess the immunological effects of branded versus generic medications.^49^ Their results indicated a notable variability in gene expression following activation by the generic GA-Natco compared to GA-Teva batches. While Copaxone effectively induced the upregulation of Treg-related genes, the GA-Natco generic was associated with increased inflammatory markers linked to monocytes and macrophages (e.g., CD14, TLR2, IL1B).^49^ It is important to note that these studies were performed in a healthy mouse model, which may not fully capture the complex dynamics of human MS. Nevertheless, these findings highlight the need for translational studies using human cells to better understand the distinctions between FOGA and Copaxone and their implications for treatment.^49,64^

The scientific discussion in the EMA public assessment report of 2016 includes a review of the above study findings, including those related to gene expression.^65^ Based on all data presented, the Board did not seek further clarification during approval of FOGAs, due to methodological issues with these studies, and concluded that Mylan GA 20 mg/mL can be regarded as therapeutically equivalent (efficacy and safety) to the reference product.^65^ Moreover, these studies notwithstanding, the EMA approved the 20 mg once-daily formulation of the Mylan FOGA in 2016,^66^ and the 40 mg thrice-weekly formulation in 2017^67^ based on the results of the GATE study, which demonstrated equivalent efficacy, safety, and tolerability of Viatris GA to the originator GA.^57^

In addition to these products, FOGA alternatives have been developed under various trade names, depending on the pharmaceutical companies that produce and distribute them (Table 1).^68,69^

**Table 1.** Glatiramoid class members approved as DMT for for relapsing MS.

GA	Manufacturer	FDA/EMA approval	Market	Scientific support to regulatory approval
Copaxone^®^	Teva	FDA and EMA	Several countries worldwide	Originator
Brabio^®^	Viatris (Legacy Mylan)	EMA	Several countries worldwide; various commercial names	Quality, preclinical, and randomized clinical data
Glatopa^®^	Sandoz	FDA	USA	No
Remurel^®^	Alvogen	EMA	Europe	No
GTR Polimunol^®^	Synthon Bagó	EMA	Argentina	No
Escadra^®^	Raffo	No	Argentina	No
Probioglat^®^	Probiomed	No	Mexico	No
Glatimer^®^	Natco	No	India	No
Copamer^®^	Zahravi	No	Iran	No
Cinnomer^®^	CinnaGen	No	Iran	NCT04928313

Source: Adapted from Annovazzi et al.^68^

DMT, disease-modifying therapy; EMA, European Medicine Agency; FDA, U.S. Food and Drug Administration; GA, glatiramer acetate.

The lack of a centralized regulatory pathway for NBCDs and their follow-on versions^70^ may affect the perception of their efficacy and safety by clinicians and patients; therefore, it is important to collect clinical data on efficacy and safety, and to conduct post-marketing surveillance to provide a better understanding of performance in real-world clinical practice. This is particularly relevant because variations in the GA production process have been associated with changes in efficacy, safety, immunogenicity, and biological activity compared to the originator GA,^51,53,59,60^ including effects on gene expression in biological pathways relevant to the pathogenesis of MS.^61–63^

This narrative review will assess the long-term safety and tolerability data for GA originator and FOGAs in the management of patients with MS. It will also present data from the Ad-hoc Safety Review Report on Viatris GA.

## GA safety

After three decades and millions of patient years, GA is still a widely prescribed DMT for treating patients with relapsing MS, despite the introduction of more efficacious DMTs.^71^ This is likely due to its ease of management, which does not require extensive monitoring, its favorable safety profile, including in special populations including older adults, patients with comorbidities, or those planning for pregnancy and breastfeeding.^72^

Currently, the longest continuous follow-up data for GA comes from the US Glatiramer Acetate Trial conducted by Ford et al., which has 27 years of safety data on 52 patients.^73,74^ The extensive phase IV clinical data collected following the initial approval of originator GA support the continuing efficacy and safety of GA in relapsing-remitting MS,^71,72^ and is used, together with post-marketing surveillance, pharmacovigilance data, scientific publications, input from expert panels, and regulatory guidance, to curate the manufacturers’ prescribing information.

Viatris, the manufacturer of a FOGA, hereafter referred to as Viatris GA, maintains a safety surveillance database of cases reported from 12 countries in Europe. Data sources include post-marketing surveys, spontaneous reports, regulatory authorities, and publications. Cumulative exposure data from Viatris for all European countries where the product has been marketed are calculated from sales data available up to March of 2022. Cumulative patient-time exposure to both of the glatiramer formulations from the launch date to June 30, 2022 is estimated at 428,168 patient years (98,955 for the 20 mg/mL dose, and 329,213 for the 40 mg/mL dose).

As of June 30, 2022, Viatris has received a total of 5366 cases from the global safety database. In 192 cases, the manufacturer of the GA product could not be established. Based on review of the cumulative data, no new safety or efficacy concerns were identified in patients switching from originator to Viatris GA, consistent with the results from patients switching from originator to Viatris GA, in the GATE Extension study,^58^ and with reported results from patients switching from originator to the FOGA Copamer^®^.^75^ Overall, cumulative post-marketing experience with Viatris GA has been consistent with the known safety profile of originator GA. More than 80% of the reported adverse events were non-serious in nature.

### Injection site reactions

Injectable MS DMTs commonly cause localized injection site reactions and a range of dermatological adverse effects, most of which are mild and, although they do not lead to discontinuation, they may impact quality of life.^76^ The most frequent treatment-emergent adverse events in pivotal trials of GA were local injection site reactions,^2,4^ consisting of transient erythema, pain, edema, and pruritus that occur with decreasing frequency over time.^77^ A pooled analysis of safety and tolerability among all MS patients exposed to GA in clinical trials before 2017 (10,017 patient-years, median exposure 1.8 years) confirmed that injection site reactions were the most common adverse events^78^; this was also consistent with the results from long-term extension studies. No late safety issues emerged in the extension studies.^4,73,74^ Injection site reactions were less frequent with the 40 mg/mL formulation administered three times weekly in the open label GLACIER study.^79^ Patients in the US Glatiramer Acetate Trial had the option of switching from daily injections to three times weekly injections when the 40 mg/mL formulation became available, but many chose to continue with daily injections, suggesting that tolerability was acceptable.^73^

### Lipoatrophy or necrosis

Injection site lipoatrophy or necrosis is a more serious adverse event that was reported in approximately 2% of patients who received GA in placebo-controlled trials, and patients should practice injection site rotation, as described in the prescribing information.^6^

### Immediate post-injection reaction

Immediate post-injection reaction may manifest as flushing, chest tightness/pain, palpitations, tachycardia, anxiety, dyspnea, throat constriction, and/or urticaria occurring within 1 h of injection. This is a rare occurrence, with a single or a few episodes occurring during long periods of treatment in 16% of treated patients.^6^ The exact nature of these reactions is unknown, and they are generally transient and self-limiting, with dyspnea occurring with the highest frequency (12%). Caution is warranted, however, as a recent direct healthcare professional communication from the EMA warns about the possibility of anaphylactic reactions occurring months to years after treatment initiation. While these reactions are uncommon (⩾1/1000 to <1/100), symptoms may overlap with those of post-injection reactions, potentially delaying their identification.^80^

### Other adverse events occurring with a frequency >10%

Consistent with findings reported in the prescribing information,^5,66^ a post hoc pooled analysis of safety and tolerability identified rashes (15%), headaches (14%), infections (12%), and episodes of vasodilation (11%).^78^ The rate of headache was similar to the reported rate of 15.8% in the general population on any given day.^81^ Infections were defined broadly, encompassing common and minor events, and the absence of a comparator arm makes interpretation difficult.^78^ Transient episodes of idiopathic chest pain, including episodes not associated with other symptoms, have also been reported. While leukopenia can be an issue with some DMTs, it occurs infrequently with GA and is generally mild if present.^82^ This is likely due to its mechanism of action, which involves anti-inflammatory cytokine shifts in CD4+ and CD8+ T cells, restoration of Tregs, and reductions in memory B and T cells.

#### Liver injury

Cases of liver injury, including hepatitis and liver failure, have been reported in patients treated with GA, occurring days to years after starting treatment.^6^ Meunier et al. have cataloged 15 cases of GA-associated liver injury, 6 of which occurred in patients who had switched to GA from interferon (IFN) due to elevated liver enzymes (Table 2).^83^ Among these 15 cases, only 4 were negative for anti-nuclear and anti-smooth muscle autoantibodies, and had not previously switched to GA due to elevated liver enzyme abnormalities associated with the previous treatment.^83^

**Table 2.** Cases of GA-associated liver injury.

Case	Sex	Age, (years)	Profile	Antibodies	Liver biopsy	Treatment before GA	Recovery (days)
Deltenre et al., 2009^84^	F	52	Hep	ANA: 1/320 ASMA: 1/80	Centrilobular damage, lymphocyte, macrophage, eosinophil	MPDN	90
Onmez et al., 2013^85^	F	36	Hep	Negative	Polymorphonuclear-rich mixed-type inflammatory cell reaction	GA + MPDN	36
Neumann et al., 2007^86^	M	71	Hep	ANA: 1/1280	Drug-induced liver injury without fibrotic changes of the liver	IFN (switch due to liver function)	30
Antezana et al., 2014^87^	F	28	Hep	Negative	Hepatocellular necrosis, portal bridging, and portal lymphocytic inflammation		30
Subramaniam et al., 2012^88^	F	31	Hep	ASMA: 1/320	Centrilobular hepatocyte necrosis with portal-venous bridging, along with mild portal and interface hepatitis		
Flaire et al., 2016^89^	F	56	Hep	Negative	Centrilobular hepatocyte necrosis with inflammatory infiltrates composed of lymphocytes and eosinophils	MPDN	45
La Gioia et al., 2014^90^	F	25	Hep	Negative	Inflammatory infiltration: lymphocytes, histiocytes, plasma cells, and a few eosinophil granulocytes		56
Makhani et al., 2013^91^	F	15	Hep	Negative	Lymphocytic inflammatory infiltration with mild portal fibrosis, no plasma cells, and no signs of chronic liver disease	IFN (switch due to liver function)	54
Fernández Fernández et al., 2015^92^	F	42	Hep	ANA: 1/640	No biopsy	IFN (switch due to liver function)	30
Sinagra et al., 2014^93^	F	41	Hep	ANA: 1/320	Moderate interface hepatitis with eosinophilic infiltration and porto-portal fibrosis	IFN (switch due to liver function)	30
Sinagra et al., 2014^93^	F	29	Hep	ANA: 1/160	Lymphoplasmacytic infiltration with porto-portal fibrosis and slight ductal proliferation	IFN (switch due to liver function)	
Almeida et al., 2017^94^	F	65	Hep	ANA: 1/40 ASMA: 1/40	No biopsy	MPDN	147
Arruti et al., 2012^95^	F	46	Hep	Negative	No biopsy		
von Kalckreuth et al., 2008^96^	F	42	Cho	ANA, ASMA positive	Severe portal and periportal lymphocytic inflammation with necrosis	IFN (switch due to liver function)	
Michels et al., 2020^97^	F	23	Hep	Negative	Hepatocyte necrosis CD38-positive lymphocytes		

Source: Reprinted from Meunier et al.^83^ Open Access under Creative Commons Attribution (CC BY) license (https://creativecommons.org/licenses/by/4.0/). No special permission is required to reuse all figures or part of article published by MDPI.

ANA, anti-nuclear antibodies; ASMA, anti-smooth muscle antibodies; Cho, cholestasis; CTC, corticosteroids; Hep, hepatitis; IFN, interferon; MPDN, methylprednisolone.

Meanwhile, Villani et al. described 11 cases of autoimmune hepatitis in untreated patients with MS,^98^ and Tsouris et al. identified similar frequencies of autoantibodies to hepatic antigens in treatment-naïve and treated patients with MS.^99^ It is important to consider the type of liver injury involved, and consider that, while case reports can provide hints about a safety signal, they require systematic assessment for confirmation.^100^

While it is not possible to estimate the frequency of drug-induced hepatotoxicity based on these cases, there were no reported cases in pivotal trials of GA originator,^101^ which may indicate that they are not common. Post-marketing surveillance in clinical practice is useful for monitoring drug safety and detecting safety signals. Spontaneous reporting systems like the FDA Adverse Event Reporting System can detect rare events like drug-induced liver injury.^102^ Disproportionality analysis of FDA Adverse Event Reporting System data acquired from 2004 through 2016 (18,591 total reports on GA) did not identify a safety signal for overall liver injury or for severe liver injury with GA, reporting odds ratios (95% CI) 0.70 (0.62–0.79), and 1.01 (0.88–1.16), respectively.^103^

In the Viatris safety surveillance database, 55 cases (65 events) suggestive of hepatic injury have been reported. Ages ranged from 23 to 90 years, and 46 patients were female. Thirty-one of 55 cases were non-serious, and 53% consisted of liver enzyme elevation. Reported forms of severe liver injury included serious drug-induced hepatotoxicity, hepatitis, autoimmune hepatitis, liver cancer, and one case of liver failure. The case of liver failure occurred in the context of multi-organ failure secondary to gastrointestinal infection, thrombosis, and pulmonary embolism, excluding a direct causal role of GA in its occurrence and fatal outcome. Of the three reported cases of autoimmune hepatitis, only one was supported by a positive anti-nuclear antibody titer, whilst information was limited in the other two. The two reported cases of liver cancer were not evaluable for causal association to GA based on limited information.

GA prescribing information recommends monitoring for signs of hepatic injury, instructing patients to seek immediate medical attention in case of symptoms of liver injury, and consider discontinuing GA in case of clinically significant liver injury.^6,67^

#### Opportunistic infections

GA modulates the immune system rather than suppressing it, promoting anti-inflammatory and regulatory pathways, while avoiding an increase in the risk of infections. DMT-associated risk of opportunistic infections is considered lowest with the injectable therapies IFN beta and GA.^78,104–106^

GA treatment is associated with a reduction of pro-inflammatory B cell functions^107^; however, leukopenia and leukocytosis are rare.^108^ Progressive multifocal leukoencephalopathy (PML), triggered by reactivation of latent John Cunningham virus, is an important safety concern when treating MS.^109^ Among DMTs, natalizumab is associated with the highest risk of PML, with rare cases also reported for ocrelizumab, fingolimod, and dimethyl fumarate. In contrast, GA has not been associated with an increased risk of PML or *hepatitis B virus* reactivation compared with the general population.^110,111^ Infection risk is a key consideration when initiating or switching treatments,^112^ and ongoing monitoring for infections is an essential practice with some DMTs.

Meanwhile, post-marketing surveillance has not shown a risk for opportunistic infections (including PML),^78,104^ and there are no requirements for infection screening or monitoring.^5^ In the Viatris database, 148 infections excluding the SARS-CoV-2 virus were reported. Of these, 75% were non-serious, and included influenza, herpes zoster, oral herpes, Staphylococcal infections, and fungal infections. Most events were consistent with the immune-modulating mechanism of action of GA, and no fatal outcomes were reported.

## Vaccination

Vaccines are not associated with an increased risk of relapses or disability in MS patients, regardless of treatment status, and their benefit greatly outweighs potential risks, as infections are frequent triggers for relapse^113–117^. Nonetheless, it may be prudent to avoid vaccination in patients who are experiencing an active relapse.^118^ Inactivated vaccines can be safely used in MS patients regardless of treatment, whereas live attenuated vaccines should be limited to untreated patients or those receiving immunomodulatory therapy with GA or IFNs.^119^

Most DMTs for MS modulate or suppress components of the immune system, potentially influencing the ability to mount an immune response to vaccines. Several DMTs are known to attenuate vaccine responses (e.g., sphingosine-1-phosphate receptor modulators, anti-CD20 monoclonal antibodies, natalizumab), and care should be taken to update vaccines before initiating these treatments, observe the optimal interval from the last DMT dose when administering annual vaccinations, and consider administering additional vaccine doses.^119^ It is recommended that vaccine-induced antibody titers should be confirmed 1–2 months after the last dose of the hepatitis B, measles, mumps, tetanus, or varicella vaccines in patients who are already receiving immunosuppressive DMTs.^119^ GA does not appear to compromise response rates to influenza, tick-born encephalitis, or SARS-CoV-2 vaccines in patients with MS.^120–123^

## Special populations

### Older adults

Older adults were not well represented in pivotal clinical studies of DMTs for MS, which generally presents in young adults^124,125^; therefore, extra attention may be warranted when prescribing DMTs in older adults, because risk–benefit considerations may change with age. This is important because as survival has improved and the population ages, the peak MS prevalence has currently increased to between 55 and 65 years.^126–128^ Disease characteristics change as patients with MS age. Older MS patients have lower relapse rates,^129^ less inflammatory activity,^130,131^ and may have less need for highly effective DMTs.^132^ Older adults with MS generally have higher rates of comorbidities, which are associated with higher risk of hospitalization,^133^ a higher risk for adverse events,^125^ and an apparent increase in MS disability progression on the Expanded Disability Status Scale.^134^ Large cohort studies have shown that DMT efficacy decreases with age,^135^ raising the question of whether de-escalation or discontinuation should be considered in older patients with no clinical or radiological disease activity. Such choices must be made in the context of shared decision-making. In a survey of older patients in the North American Research Committee on Multiple Sclerosis Registry who had been receiving the same DMT for at least 5 years, 88.1% of 377 respondents reported being unlikely to consider DMT discontinuation if their MS was stable^136^; therefore, de-escalation may be a more attractive option to discontinuation in this situation. Moreover, the results of the DISCOMS (discontinue disease-modifying therapies) and DOT-MS studies do not support the discontinuation of DMTs in older patients.^137,138^ However, the DISCOMS study had a non-inferiority design, and the difference was very small at 7.5 percentage points.^137^ Thus, decisions should be made on an individual basis considering impact on quality of life and patient preference.^139^

### Patients with comorbidities and polypharmacy

Meta-analysis of 249 studies on comorbidities in MS identified depression, anxiety, hypertension, hypercholesterolemia, and chronic lung disease as the most prevalent comorbidities overall,^140^ while the most prevalent autoimmune comorbidities were thyroid disease and psoriasis.^141^ Number of comorbidities may also impact the choice of MS DMT favoring GA.^142^ Patients with comorbid systemic autoimmune conditions may benefit from GA as a treatment option. Clinical trials investigating the potential benefit in other autoimmune disorders are not available. There is only a case series about nine patients with MS and systemic lupus erythematosus, two of whom were treated with GA and experienced no exacerbation of the lupus-associated symptoms.^143^ In another case series, successful treatment of uveitis with GA was reported.^144^

Administering DMTs for MS, together with symptomatic drugs and therapy for comorbid conditions can result in polypharmacy,^145,146^ which has been shown to increase the risk of pharmacodynamic and pharmacokinetic drug–drug interactions in patients with MS.^147^ Drug–drug interactions can cause side effects or alter the effectiveness of drugs, increasing the risk of treatment failure. In addition to toxicity, polypharmacy may be associated with a higher risk of falls in patients with MS, particularly in patients receiving anti-cholinergic agents,^148^ as well as fatigue and cognitive impairment.^149^ A useful strategy for avoiding this includes identifying potential interactions using a database (e.g., Hecker et al.^150^), followed by elimination of unnecessary drugs and supplements, and considering alternative agents with lower potential for interaction, when possible.

Drug interactions involving GA do not appear to be well studied; however, results from clinical trials do not suggest significant interactions with therapies commonly used in MS patients, including concurrent corticosteroids for up to 1 month.^5,6^

### Use in pregnancy

Pregnancy is associated with reduced relapse rates and may be followed by a post-partum rebound of relapse frequency during the months after birth.^151–156^ Safety during pregnancy is a critical issue for MS treatment with DMTs, because many of the patients are women of childbearing age. Treatment decisions around pregnancy must consider prior disease activity and whether a woman is receiving DMTs with a high risk of disease rebound after discontinuation.^157^

For this reason, several database studies have assessed the safety of GA during pregnancy. Teva’s global pharmacovigilance database (*n* = 5042 pregnancies with known outcomes) revealed an incidence of congenital anomalies that was compatible with the expected rate based on large population studies.^158,159^ A systematic review and meta-analysis of pregnancy and fetal outcomes in women receiving GA (or IFN or natalizumab) did not reveal an increase in the risk of spontaneous abortions, preterm birth, or major congenital malformations.^160^ Meanwhile, analysis of data from the German Multiple Sclerosis and Pregnancy Registry also showed rates of such outcomes similar to those in the general population.^161^ Exposure to GA (20 or 40 mg formulations) throughout all three pregnancy trimesters was not associated with adverse pregnancy or infant outcomes.^162^

In the Viatris database, 445 exposed pregnancies were reported. Common adverse outcomes related to pregnancy included 23 instances of (spontaneous) abortion, 7 preterm births, 3 ectopic pregnancies, 3 reports of vanishing twin syndrome, and 2 cases of oligohydramnios. Congenital malformations were reported in seven cases (1.6%); however, based on the nature of post-marketing data and the evidence available from these cases, it is not possible to determine if this incidence differs from that in unexposed pregnancies. Both originator and Viatris GA can be used in pregnancy.^6,66^

### Breastfeeding

Breastfeeding is advantageous for the infant and may be associated with a reduction in postpartum MS relapses^163^; however, there are concerns about breastfeeding while receiving DMTs. Both originator GA and Viatris GA can be used during breastfeeding.^6,66^ Data from the German MS and Pregnancy Registry did not reveal evidence of adverse effects of GA exposure during breastfeeding on infant development, hospitalization, or the use of antibiotics.^164–166^ Maternal use of GA during breastfeeding did not adversely affect offspring safety outcomes assessed during the first 18 months of life. Published pharmacovigilance data on exposed pregnancies confirm the lack of adverse outcomes with originator GA during breastfeeding.^159^

The use of Viatris GA during breastfeeding was reported in 60 cases in the Viatris database. Concurrent events reported were either injection site reactions or device or administration-related issues. No adverse events were identified in the infants that could be attributed to exposure to GA via breast milk. Currently, there are no regulatory restrictions on GA use while breastfeeding.^6,67^

### Patients at risk for infections and reactivation of latent infections

Individuals at increased risk of infections include those receiving immunosuppressive medications to prevent rejection of a transplanted organ, patients with HIV, oncology patients who receive chemotherapy or radiation therapy, and older adults experiencing immunosenescence. Infections contribute substantially to morbidity and mortality in MS.^108,167,168^ Compared with the general population, people with MS have approximately twice the incidence of infections and infection-related hospitalizations,^169^ most frequently involving the respiratory^170^ and urinary tracts.^171^ Immunosuppressive therapies may further increase infection risk.^172^ All DMTs for MS, with the exception of IFNs and GA, can impair immune surveillance.^104,105^

Patients with MS do not appear to be at higher risk of SARS-CoV-2 infection compared to healthy subjects^173^; however, the rate of COVID-19 development in patients with MS is lower in those receiving GA (0.51%) or IFNs (0.61%), compared to other DMTs (*p* < 0.001 for both), whereas anti-CD20 therapies are associated with the highest risk (3.45%; *p* < 0.0001).^174^ Pretreatment screening for latent infections is not required with GA.^6^

## Conclusion

The many effective DMTs now available have improved the personalization of treatment for MS. Extensive clinical experience continues to demonstrate the enduring value of GA in the management of relapsing MS not requiring a highly effective DMT. Ease of management, good tolerability, and long-term safety data make GA an appropriate choice for diverse patient populations, including patients with comorbidities, breastfeeding individuals, older adults, and patients undergoing therapeutic de-escalation. A minimal impact on immune function makes GA a suitable option for MS patients at risk of infections, while compatibility with vaccinations ensures that essential immunizations will be effective. Localized injection site reactions, although common, do not typically lead to treatment discontinuation. More severe injection site events like lipoatrophy or necrosis are rare, and while there are reported cases of liver injury, their frequency remains low. Available evidence on product substitution from branded to FOGAs shows similar efficacy and safety reinforcing its position as a reliable and enduring therapeutic option for MS management.

## Declarations

**Ethics approval and consent to participate:** Not applicable.

**Consent for publication:** Not applicable.

**Competing interests:** R.P. received honoraria for lecturing and consulting from Alexion, Bayer Healthcare, Biogen, Bristol Myers Squibb, Horizon/Amgen, MedDay, Merck Serono, Novartis, Roche, Sanofi Genzyme/Aventis, and Viatris. He received research funds from Teva, Merck Serono, Bristol Myers Squibb and Novartis. J.S. received lecture fees from Merck and Sanofi, and travel grants from Novartis and Sanofi. C.K. received honoraria for lecturing and consulting as well as financial research support from Ablynx, Almirall, Amgen, Bayer Vital, Bristol Myers Squibb, Biotronik, Boehringer Ingelheim, Biogen, CSL Behring, Daiichi-Sankyo, Desitin, Eisai, Ever Pharma, Sanofi Genzyme, Merck Serono, Mylan, Medday, Novartis, Pfizer, Roche, Siemens, Stago, Teva, and Viatris. S.B.S. and R.A.S. are employees of Viatris.

**Availability of data and materials:** Not applicable.

## References

[1] Bornstein MB Miller AI Teitelbaum D , et al. Multiple sclerosis: trial of a synthetic polypeptide. Ann Neurol 1982; 11: 317–319. 7092185 10.1002/ana.410110314

[2] Johnson KP Brooks BR Cohen JA , et al. Copolymer 1 reduces relapse rate and improves disability in relapsing-remitting multiple sclerosis: results of a phase III multicenter, double-blind placebo-controlled trial. The Copolymer 1 Multiple Sclerosis Study Group. Neurology 1995; 45: 1268–1276. 7617181 10.1212/wnl.45.7.1268

[3] Johnson KP Brooks BR Cohen JA , et al. Extended use of glatiramer acetate (Copaxone) is well tolerated and maintains its clinical effect on multiple sclerosis relapse rate and degree of disability. Copolymer 1 Multiple Sclerosis Study Group. Neurology 1998; 50: 701–708. 9521260 10.1212/wnl.50.3.701

[4] Comi G Filippi M Wolinsky JS. European/Canadian multicenter, double-blind, randomized, placebo-controlled study of the effects of glatiramer acetate on magnetic resonance imaging-measured disease activity and burden in patients with relapsing multiple sclerosis. European/Canadian Glatiramer Acetate Study Group. Ann Neurol 2001; 49: 290–297. 11261502

[5] Teva Neuroscience. Copaxone prescribing information, https://www.copaxone.com/globalassets/copaxone/prescribing-information.pdf (2023, accessed 30 May 2023).

[6] Teva. Copaxone—summary of product characteristics. Electronic medicines compendium, https://www.medicines.org.uk/emc/product/183 (2022, accessed 11 October 2023).

[7] Teitelbaum D Meshorer A Hirshfeld T , et al. Suppression of experimental allergic encephalomyelitis by a synthetic polypeptide. Eur J Immunol 1971; 1: 242–248. 5157960 10.1002/eji.1830010406

[8] Lalive PH Neuhaus O Benkhoucha M , et al. Glatiramer acetate in the treatment of multiple sclerosis: emerging concepts regarding its mechanism of action. CNS Drugs 2011; 25: 401–414. 21476611 10.2165/11588120-000000000-00000 PMC3963480

[9] Borchard G Crommelin DJA . Equivalence of glatiramer acetate products: challenges in assessing pharmaceutical equivalence and critical clinical performance attributes. Expert Opin Drug Deliv 2018; 15: 247–259. 29241378 10.1080/17425247.2018.1418322

[10] Rocco P Eberini I Musazzi UM , et al. Glatiramer acetate: a complex drug beyond biologics. Eur J Pharm Sci 2019; 133: 8–14. 30902653 10.1016/j.ejps.2019.03.011

[11] Aharoni R Teitelbaum D Sela M , et al. Bystander suppression of experimental autoimmune encephalomyelitis by T cell lines and clones of the Th2 type induced by copolymer 1. J Neuroimmunol 1998; 91: 135–146. 9846830 10.1016/s0165-5728(98)00166-0

[12] Haas J Korporal M Balint B , et al. Glatiramer acetate improves regulatory T-cell function by expansion of naive CD4(+)CD25(+)FOXP3(+)CD31(+) T-cells in patients with multiple sclerosis. J Neuroimmunol 2009; 216: 113–117. 19646767 10.1016/j.jneuroim.2009.06.011

[13] Aharoni R Eilam R Stock A , et al. Glatiramer acetate reduces Th-17 inflammation and induces regulatory T-cells in the CNS of mice with relapsing-remitting or chronic EAE. J Neuroimmunol 2010; 225: 100–111. 20554028 10.1016/j.jneuroim.2010.04.022

[14] Oreja-Guevara C Ramos-Cejudo J Aroeira LS , et al. TH1/TH2 Cytokine profile in relapsing-remitting multiple sclerosis patients treated with Glatiramer acetate or Natalizumab. BMC Neurol 2012; 12: 95. 22989378 10.1186/1471-2377-12-95 PMC3517482

[15] Pul R Morbiducci F Škuljec J , et al. Glatiramer acetate increases phagocytic activity of human monocytes in vitro and in multiple sclerosis patients. PLoS One 2012; 7: e51867. 10.1371/journal.pone.0051867 PMC3527448 23284793

[16] Weber MS Prod’homme T Youssef S , et al. Type II monocytes modulate T cell-mediated central nervous system autoimmune disease. Nat Med 2007; 13: 935–943. 17676050 10.1038/nm1620

[17] Kuerten S Jackson LJ Kaye J , et al. Impact of glatiramer acetate on B cell-mediated pathogenesis of multiple sclerosis. CNS Drugs 2018; 32: 1039–1051. 30315499 10.1007/s40263-018-0567-8 PMC6223706

[18] Sand KL Knudsen E Rolin J , et al. Modulation of natural killer cell cytotoxicity and cytokine release by the drug glatiramer acetate. Cell Mol Life Sci 2009; 66: 1446–1456. 19277466 10.1007/s00018-009-8726-1 PMC11131507

[19] Al-Falahi Y Sand KL Knudsen E , et al. Splenic natural killer cell activity in two models of experimental neurodegenerative diseases. J Cell Mol Med 2009; 13: 2693–2703. 19397784 10.1111/j.1582-4934.2008.00640.x PMC6529976

[20] Høglund RA Holmøy T Harbo HF , et al. A one year follow-up study of natural killer and dendritic cells activities in multiple sclerosis patients receiving glatiramer acetate (GA). PLoS One 2013; 8: e62237. 10.1371/journal.pone.0062237 PMC3632560 23614042

[21] Maghazachi AA Sand KL Al-Jaderi Z. Glatiramer acetate, dimethyl fumarate, and monomethyl fumarate upregulate the expression of CCR10 on the surface of natural killer cells and enhance their chemotaxis and cytotoxicity. Front Immunol 2016; 7: 437. 27807435 10.3389/fimmu.2016.00437 PMC5069502

[22] Sellner J Koczi W Harrer A , et al. Glatiramer acetate attenuates the pro-migratory profile of adhesion molecules on various immune cell subsets in multiple sclerosis. Clin Exp Immunol 2013; 173: 381–389. 23611040 10.1111/cei.12125 PMC3949625

[23] Amrouche K Pers J-O Jamin C. Glatiramer acetate stimulates regulatory B cell functions. J Immunol 2019; 202: 1970–1980. 30745460 10.4049/jimmunol.1801235

[24] Häusler D Hajiyeva Z Traub JW , et al. Glatiramer acetate immune modulates B-cell antigen presentation in treatment of MS. Neurol Neuroimmunol Neuroinflamm 2020; 7: e698. 10.1212/NXI.0000000000000698 PMC7136047 32184341

[25] Jackson DG. Lymphatic regulation of cellular trafficking. J Clin Cell Immunol 2014; 5: 258. 27808282 10.4172/2155-9899.1000258 PMC5081098

[26] Carrieri PB Carbone F Perna F , et al. Longitudinal assessment of immuno-metabolic parameters in multiple sclerosis patients during treatment with glatiramer acetate. Metabolism 2015; 64: 1112–1121. 25986733 10.1016/j.metabol.2015.05.001

[27] Sellebjerg F Hedegaard CJ Krakauer M , et al. Glatiramer acetate antibodies, gene expression and disease activity in multiple sclerosis. Mult Scler 2012; 18: 305–313. 22020419 10.1177/1352458511420268

[28] Harp CT Ireland S Davis LS , et al. Memory B cells from a subset of treatment-naïve relapsing-remitting multiple sclerosis patients elicit CD4(+) T-cell proliferation and IFN-γ production in response to myelin basic protein and myelin oligodendrocyte glycoprotein. Eur J Immunol 2010; 40: 2942–2956. 20812237 10.1002/eji.201040516 PMC3072802

[29] Vieira PL Heystek HC Wormmeester J , et al. Glatiramer acetate (copolymer-1, copaxone) promotes Th2 cell development and increased IL-10 production through modulation of dendritic cells. J Immunol 2003; 170: 4483–4488. 12707324 10.4049/jimmunol.170.9.4483

[30] Jung S Siglienti I Grauer O , et al. Induction of IL-10 in rat peritoneal macrophages and dendritic cells by glatiramer acetate. J Neuroimmunol 2004; 148: 63–73. 14975587 10.1016/j.jneuroim.2003.11.014

[31] Kim HJ Ifergan I Antel JP , et al. Type 2 monocyte and microglia differentiation mediated by glatiramer acetate therapy in patients with multiple sclerosis. J Immunol 2004; 172: 7144–7153. 15153538 10.4049/jimmunol.172.11.7144

[32] Weber MS Starck M Wagenpfeil S , et al. Multiple sclerosis: glatiramer acetate inhibits monocyte reactivity in vitro and in vivo. Brain 2004; 127: 1370–1378. 15090474 10.1093/brain/awh163

[33] Burger D Molnarfi N Weber MS , et al. Glatiramer acetate increases IL-1 receptor antagonist but decreases T cell-induced IL-1beta in human monocytes and multiple sclerosis. Proc Natl Acad Sci U S A 2009; 106: 4355–4359. 19255448 10.1073/pnas.0812183106 PMC2649955

[34] Carpintero R Brandt KJ Gruaz L , et al. Glatiramer acetate triggers PI3Kδ/Akt and MEK/ERK pathways to induce IL-1 receptor antagonist in human monocytes. Proc Natl Acad Sci U S A 2010; 107: 17692–17697. 20876102 10.1073/pnas.1009443107 PMC2955124

[35] Chabot S Yong FP Le DM , et al. Cytokine production in T lymphocyte-microglia interaction is attenuated by glatiramer acetate: a mechanism for therapeutic efficacy in multiple sclerosis. Mult Scler 2002; 8: 299–306. 12166500 10.1191/1352458502ms810oa

[36] Molnarfi N Prod’homme T Schulze-Topphoff U , et al. Glatiramer acetate treatment negatively regulates type I interferon signaling. Neurol Neuroimmunol Neuroinflamm 2015; 2: e179. 10.1212/NXI.0000000000000179 PMC4645172 26601118

[37] Kasindi A Fuchs D-T Koronyo Y , et al. Glatiramer acetate immunomodulation: evidence of neuroprotection and cognitive preservation. Cells 2022; 11: 1578. 35563884 10.3390/cells11091578 PMC9099707

[38] Aharoni R Arnon R Eilam R. Neurogenesis and neuroprotection induced by peripheral immunomodulatory treatment of experimental autoimmune encephalomyelitis. J Neurosci 2005; 25: 8217–8228. 16148229 10.1523/JNEUROSCI.1859-05.2005 PMC6725544

[39] Aharoni R. Immunomodulation neuroprotection and remyelination—the fundamental therapeutic effects of glatiramer acetate: a critical review. J Autoimmun 2014; 54: 81–92. 24934599 10.1016/j.jaut.2014.05.005

[40] Arnon R Aharoni R. Neurogenesis and neuroprotection in the CNS-fundamental elements in the effect of Glatiramer acetate on treatment of autoimmune neurological disorders. Mol Neurobiol 2007; 36: 245–253. 17955199 10.1007/s12035-007-8002-z

[41] Skihar V Silva C Chojnacki A , et al. Promoting oligodendrogenesis and myelin repair using the multiple sclerosis medication glatiramer acetate. Proc Natl Acad Sci U S A 2009; 106: 17992–17997. 19815532 10.1073/pnas.0909607106 PMC2758287

[42] Gentile A Rossi S Studer V , et al. Glatiramer acetate protects against inflammatory synaptopathy in experimental autoimmune encephalomyelitis. J Neuroimmune Pharmacol 2013; 8: 651–663. 23370991 10.1007/s11481-013-9436-x

[43] Aharoni R Eilam R Lerner S , et al. Neuroprotective effect of glatiramer acetate on neurofilament light chain leakage and glutamate excess in an animal model of multiple sclerosis. Int J Mol Sci 2021; 22: 13419. 34948217 10.3390/ijms222413419 PMC8707261

[44] Multiple Sclerosis International Federation. Atlas of MS—3rd Edition, https://www.atlasofms.org/chart/united-kingdom/disease-modifying-treatments/barriers-to-accessing-dmts (2023, accessed 29 July 2025).

[45] Moss BP Cohen JA. The emergence of follow-on disease-modifying therapies for multiple sclerosis. Mult Scler 2019; 25: 1560–1565. 31074707 10.1177/1352458519845106

[46] Hartung DM. Economics of multiple sclerosis disease-modifying therapies in the USA. Curr Neurol Neurosci Rep 2021; 21: 28. 33948740 10.1007/s11910-021-01118-x

[47] Alton G Samuel C Reddi A. Providing access to high-quality, low-cost medicines across low and middle-income countries (LMICs), working with governments and generic manufacturers around the globe—a business case. Antivir Ther 2022; 27: 13596535211068617. 10.1177/13596535211068617 35491613

[48] Greenberg B Giovannoni G. A place for biosimilars in the changing multiple sclerosis treatment landscape. Mult Scler Relat Disord 2023; 77: 104841. 37467536 10.1016/j.msard.2023.104841

[49] Towfic F Funt JM Fowler KD , et al. Comparing the biological impact of glatiramer acetate with the biological impact of a generic. PLoS One 2014; 9: e83757. 10.1371/journal.pone.0083757 PMC3885444 24421904

[50] D’Alessandro JS Duffner J Pradines J , et al. Equivalent Gene Expression Profiles between Glatopa™ and Copaxone^®^. PLoS One 2015; 10: e0140299. 10.1371/journal.pone.0140299 PMC4608686 26473741

[51] Rogstad S Pang E Sommers C , et al. Modern analytics for synthetically derived complex drug substances: NMR, AFFF-MALS, and MS tests for glatiramer acetate. Anal Bioanal Chem 2015; 407: 8647–8659. 26458562 10.1007/s00216-015-9057-8

[52] D’ Alessandro J Garofalo K Zhao G , et al. Demonstration of biological and immunological equivalence of a generic glatiramer acetate. CNS Neurol Disord Drug Targets 2017; 16: 714–723. 28240190 10.2174/1871527316666170223162747 PMC5684786

[53] Melamed-Gal S Loupe P Timan B , et al. Physicochemical, biological, functional and toxicological characterization of the European follow-on glatiramer acetate product as compared with Copaxone. eNeurologicalSci 2018; 12: 19–30. 30094354 10.1016/j.ensci.2018.05.006 PMC6073084

[54] Arends RJ Wang D Buurman M , et al. Comparison of Copaxone® and Synthon’s therapeutically equivalent glatiramer acetate. Pharmazie 2019; 74: 449–461. 31526436 10.1691/ph.2019.9515

[55] Gaspar RS Silva-Lima B Magro F , et al. Non-biological complex drugs (NBCDs): complex pharmaceuticals in need of individual robust clinical assessment before any therapeutic equivalence decision. Front Med (Lausanne) 2020; 7: 590527. 33330550 10.3389/fmed.2020.590527 PMC7719831

[56] Anderson J Bell C Bishop J , et al. Demonstration of equivalence of a generic glatiramer acetate (Glatopa™). J Neurol Sci 2015; 359: 24–34. 26671082 10.1016/j.jns.2015.10.007

[57] Cohen J Belova A Selmaj K , et al. Equivalence of generic glatiramer acetate in multiple sclerosis: a randomized clinical trial. JAMA Neurol 2015; 72: 1433–1441. 26458034 10.1001/jamaneurol.2015.2154

[58] Selmaj K Barkhof F Belova AN , et al. Switching from branded to generic glatiramer acetate: 15-month GATE trial extension results. Mult Scler 2017; 23: 1909–1917. 28090798 10.1177/1352458516688956 PMC5700775

[59] Varkony H Weinstein V Klinger E , et al. The glatiramoid class of immunomodulator drugs. Expert Opin Pharmacother 2009; 10: 657–668. 19245345 10.1517/14656560902802877

[60] Schellekens H Stegemann S Weinstein V , et al. How to regulate nonbiological complex drugs (NBCD) and their follow-on versions: points to consider. AAPS J 2014; 16: 15–21. 24065600 10.1208/s12248-013-9533-z PMC3889532

[61] Hasson T Kolitz S Towfic F , et al. Functional effects of the antigen glatiramer acetate are complex and tightly associated with its composition. J Neuroimmunol 2016; 290: 84–95. 26711576 10.1016/j.jneuroim.2015.11.020

[62] Bakshi S Chalifa-Caspi V Plaschkes I , et al. Gene expression analysis reveals functional pathways of glatiramer acetate activation. Expert Opin Ther Targets 2013; 17: 351–362. 23469939 10.1517/14728222.2013.778829

[63] Kolitz S Hasson T Towfic F , et al. Gene expression studies of a human monocyte cell line identify dissimilarities between differently manufactured glatiramoids. Sci Rep 2015; 5: 10191. 25998228 10.1038/srep10191 PMC4441120

[64] Seok J Warren HS Cuenca AG , et al. Genomic responses in mouse models poorly mimic human inflammatory diseases. Proc Natl Acad Sci U S A. 2013; 110(9): 3507–3512. 23401516 10.1073/pnas.1222878110 PMC3587220

[65] Dutch Medicines Authority CBG. Public Assessment Report Scientific discussion Glatirameeracetaat Mylan 20 mg/ml, solution for injection (glatiramer acetate) NL/H/3213/001/DC, https://db.cbg-meb.nl/pars/h115993.pdf (2016).

[66] Viatris. Brabio 20 mg/mL—summary of product characteristics. Electronic Medicines Compendium, https://www.medicines.org.uk/emc/product/8536 (2022, accessed 11 October 2023).

[67] Viatris. Brabio 40 mg/mL—summary of product characteristics. Electronic Medicines Compendium, https://www.medicines.org.uk/emc/product/9181/smpc#gref (2022, accessed 11 October 2023).

[68] Annovazzi P Bertolotto A Brescia Morra V , et al. A comprehensive review on Copemyl®. Neurol Ther 2017; 6: 161–173. 28762192 10.1007/s40120-017-0079-3 PMC5700901

[69] Bell C Anderson J Ganguly T , et al. Development of Glatopa® (Glatiramer Acetate): the first FDA-approved generic disease-modifying therapy for relapsing forms of multiple sclerosis. J Pharm Pract 2018; 31: 481–488. 28847230 10.1177/0897190017725984 PMC6144347

[70] Klein K Stolk P De Bruin ML , et al. The EU regulatory landscape of non-biological complex drugs (NBCDs) follow-on products: observations and recommendations. Eur J Pharm Sci 2019; 133: 228–235. 30953753 10.1016/j.ejps.2019.03.029

[71] Wynn DR. Enduring clinical value of Copaxone® (glatiramer acetate) in multiple sclerosis after 20 years of use. Mult Scler Int 2019; 2019: 7151685. 30775037 10.1155/2019/7151685 PMC6350531

[72] Mirabella M Annovazzi P Brownlee W , et al. Treatment challenges in multiple sclerosis—a continued role for glatiramer acetate? Front Neurol 2022; 13: 844873. 35493825 10.3389/fneur.2022.844873 PMC9051342

[73] Ford C Goodman AD Johnson K , et al. Continuous long-term immunomodulatory therapy in relapsing multiple sclerosis: results from the 15-year analysis of the US prospective open-label study of glatiramer acetate. Mult Scler 2010; 16: 342–350. 20106943 10.1177/1352458509358088 PMC2850588

[74] Ford CC Cohen JA Goodman AD , et al. Early versus delayed treatment with glatiramer acetate: analysis of up to 27 years of continuous follow-up in a US open-label extension study. Mult Scler 2022; 28: 1729–1743. 35768939 10.1177/13524585221094239 PMC9442630

[75] Abolfazli R Pournourmohammadi S Shamshiri A , et al. Tolerability and safety profile of a new brand-generic product of glatiramer acetate in iranian patients with relapsing-remitting multiple sclerosis: an observational cohort study. Curr Ther Res Clin Exp 2018; 88: 47–51. 29928468 10.1016/j.curtheres.2018.05.001 PMC6008499

[76] Balak DMW Hengstman GJD Çakmak A , et al. Cutaneous adverse events associated with disease-modifying treatment in multiple sclerosis: a systematic review. Mult Scler 2012; 18: 1705–1717. 22371220 10.1177/1352458512438239

[77] Ziemssen T Neuhaus O Hohlfeld R. Risk-benefit assessment of glatiramer acetate in multiple sclerosis. Drug Saf 2001; 24: 979–990. 11735654 10.2165/00002018-200124130-00005

[78] Ziemssen T Ashtamker N Rubinchick S , et al. Long-term safety and tolerability of glatiramer acetate 20 mg/ml in the treatment of relapsing forms of multiple sclerosis. Expert Opin Drug Saf 2017; 16: 247–255. 27989217 10.1080/14740338.2017.1274728

[79] Wolinsky JS Borresen TE Dietrich DW , et al. GLACIER: an open-label, randomized, multicenter study to assess the safety and tolerability of glatiramer acetate 40 mg three-times weekly versus 20 mg daily in patients with relapsing-remitting multiple sclerosis. Mult Scler Relat Disord 2015; 4: 370–376. 26195058 10.1016/j.msard.2015.06.005

[80] European Medicines Agency. Direct Healthcare Professional Communication (DHPC) Glatiramer acetate: anaphylactic reactions may occur months up to years after treatment initiation, https://www.ema.europa.eu/en/documents/dhpc/direct-healthcare-professional-communication-dhpc-glatiramer-acetate-anaphylactic-reactions-may-occur-months-years-after-treatment-initiation_en.pdf (2024).

[81] Stovner LJ Hagen K Linde M , et al. The global prevalence of headache: an update, with analysis of the influences of methodological factors on prevalence estimates. J Headache Pain 2022; 23: 34. 35410119 10.1186/s10194-022-01402-2 PMC9004186

[82] Winkelmann A Loebermann M Reisinger EC , et al. Multiple sclerosis treatment and infectious issues: update 2013. Clin Exp Immunol 2014; 175: 425–438. 24134716 10.1111/cei.12226 PMC3927903

[83] Meunier L Larrey D. Hepatotoxicity of drugs used in multiple sclerosis, diagnostic challenge, and the role of HLA genotype susceptibility. Int J Mol Sci 2023; 24: 852. 36614299 10.3390/ijms24010852 PMC9821303

[84] Deltenre P Peny M-O Dufour A , et al. Acute hepatitis induced by glatiramer acetate. BMJ Case Rep 2009; 2009: bcr09.2008.0913. 10.1136/bcr.09.2008.0913 PMC3027796 21686565

[85] Onmez A Eminler AT Ergenç H , et al. Drug-induced liver injury by glatiramer acetate used for treatment of multiple sclerosis: a case report. J Investig Med High Impact Case Rep 2013; 1: 2324709613517493. 10.1177/2324709613517493 PMC4528838 26425591

[86] Neumann H Csepregi A Sailer M , et al. Glatiramer acetate induced acute exacerbation of autoimmune hepatitis in a patient with multiple sclerosis. J Neurol 2007; 254: 816–817. 17351724 10.1007/s00415-006-0441-3

[87] Antezana A Herbert J Park J , et al. Glatiramer acetate-induced acute hepatotoxicity in an adolescent with MS. Neurology 2014; 82: 1846–1847. 10.1212/01.wnl.0000450224.37865.80 24843037

[88] Subramaniam K Pavli P Llewellyn H , et al. Glatiramer acetate induced hepatotoxicity. Curr Drug Saf 2012; 7: 186–188. 22873505 10.2174/157488612802715690

[89] Flaire A Carra-Dalliere C Ayrignac X , et al. Glatiramer acetate-induced hepatitis in a patient with multiple sclerosis. Acta Neurol Belg 2016; 116: 99–100. 26141739 10.1007/s13760-015-0506-0

[90] La Gioia S Bacis G Sonzogni A , et al. Glatiramer acetate-induced hepatitis in a young female patient with multiple sclerosis. Mult Scler Relat Disord 2014; 3: 732–734. 25891553 10.1016/j.msard.2014.08.001

[91] Makhani N Ngan B Kamath BM , et al. Glatiramer acetate-induced acute hepatotoxicity in an adolescent with MS. Neurology 2013; 81: 850–852. 23884038 10.1212/WNL.0b013e3182a2cc4a PMC3908464

[92] Fernández Fernández N Joao Matias D Pisabarros Blanco C , et al. [Hepatitis induced by glatiramer acetate]. Gastroenterol Hepatol 2015; 38: 280–281. 25042243 10.1016/j.gastrohep.2014.06.001

[93] Sinagra E Raimondo D Cottone S , et al. Does glatiramer acetate provoke hepatitis in multiple sclerosis? Mult Scler Relat Disord 2014; 3: 266–268. 25878015 10.1016/j.msard.2013.09.008

[94] Almeida J Solà-Valls N Pose E , et al. Liver injury and glatiramer acetate, an uncommon association: case report and literature review. Ther Adv Neurol Disord 2017; 10: 367–372. 29090021 10.1177/1756285617722352 PMC5642009

[95] Arruti M Castillo-Triviño T de la Riva P , et al. [Autoimmune hepatitis in a patient with multiple sclerosis under treatment with glatiramer acetate]. Rev Neurol 2012; 55: 190–192. 22825980

[96] von Kalckreuth V Lohse AW Schramm C. Unmasking autoimmune hepatitis under immunomodulatory treatment of multiple sclerosis—not only beta interferon. Am J Gastroenterol 2008; 103: 2147–2148; author reply 2148. 10.1111/j.1572-0241.2008.01982_9.x 18796115

[97] Michels S Zizer E Barth TF , et al. Drug-induced liver injury associated with the biosimilar glatiramer acetate (Clift®). Mult Scler Relat Disord 2020; 40: 101948. 31972518 10.1016/j.msard.2020.101948

[98] Villani R Serviddio G Avolio C , et al. Autoimmune liver disease and multiple sclerosis: state of the art and future perspectives. Clin Exp Med. 2023; 23(7): 3321–3338. 37421590 10.1007/s10238-023-01128-8 PMC10618321

[99] Tsouris Z Liaskos C Dardiotis E , et al. A comprehensive analysis of antigen-specific autoimmune liver disease related autoantibodies in patients with multiple sclerosis. Auto Immun Highlights 2020; 11: 7. 32308974 10.1186/s13317-020-00130-4 PMC7147023

[100] Björnsson ES Hoofnagle JH. Categorization of drugs implicated in causing liver injury: critical assessment based on published case reports. Hepatology 2016; 63: 590–603. 26517184 10.1002/hep.28323

[101] Biolato M Bianco A Lucchini M , et al. The disease-modifying therapies of relapsing-remitting multiple sclerosis and liver injury: a narrative review. CNS Drugs 2021; 35: 861–880. 34319570 10.1007/s40263-021-00842-9 PMC8354931

[102] US Food and Drug Administration. FDA adverse event reporting system, https://www.fda.gov/drugs/questions-and-answers-fdas-adverse-event-reporting-system-faers/fda-adverse-event-reporting-system-faers-latest-quarterly-data-files (2023, accessed 29 July 2025).

[103] Antonazzo IC Poluzzi E Forcesi E , et al. Liver injury with drugs used for multiple sclerosis: a contemporary analysis of the FDA Adverse Event Reporting System. Mult Scler 2019; 25: 1633–1640. 30230957 10.1177/1352458518799598

[104] Wijnands JMA Zhu F Kingwell E , et al. Disease-modifying drugs for multiple sclerosis and infection risk: a cohort study. J Neurol Neurosurg Psychiatry 2018; 89: 1050–1056. 29602795 10.1136/jnnp-2017-317493

[105] Luna G Alping P Burman J , et al. Infection risks among patients with multiple sclerosis treated with fingolimod, natalizumab, rituximab, and injectable therapies. JAMA Neurol 2020; 77: 184–191. 31589278 10.1001/jamaneurol.2019.3365 PMC6784753

[106] Tur C Dubessy A-L Otero-Romero S , et al. The risk of infections for multiple sclerosis and neuromyelitis optica spectrum disorder disease-modifying treatments: Eighth European Committee for Treatment and Research in Multiple Sclerosis Focused Workshop Review. April 2021. Mult Scler 2022; 28: 1424–1456. 10.1177/13524585211069068 35196927

[107] Ireland SJ Guzman AA O’Brien DE , et al. The effect of glatiramer acetate therapy on functional properties of B cells from patients with relapsing-remitting multiple sclerosis. JAMA Neurol 2014; 71: 1421–1428. 25264704 10.1001/jamaneurol.2014.1472 PMC4335670

[108] Winkelmann A Loebermann M Reisinger EC , et al. Disease-modifying therapies and infectious risks in multiple sclerosis. Nat Rev Neurol 2016; 12: 217–233. 26943779 10.1038/nrneurol.2016.21

[109] Cortese I Reich DS Nath A. Progressive multifocal leukoencephalopathy and the spectrum of JC virus-related disease. Nat Rev Neurol 2021; 17: 37–51. 33219338 10.1038/s41582-020-00427-y PMC7678594

[110] Berger JR. Classifying PML risk with disease modifying therapies. Mult Scler Relat Disord 2017; 12: 59–63. 28283109 10.1016/j.msard.2017.01.006

[111] Perrillo RP Gish R Falck-Ytter YT. American Gastroenterological Association Institute technical review on prevention and treatment of hepatitis B virus reactivation during immunosuppressive drug therapy. Gastroenterology 2015; 148: 221-244.e3. 10.1053/j.gastro.2014.10.038 25447852

[112] Celius EG. Infections in patients with multiple sclerosis: implications for disease-modifying therapy. Acta Neurol Scand 2017; 136 Suppl: 34–36. 10.1111/ane.12835 29068490

[113] Kappos L Mehling M Arroyo R , et al. Randomized trial of vaccination in fingolimod-treated patients with multiple sclerosis. Neurology 2015; 84: 872–879. 25636714 10.1212/WNL.0000000000001302

[114] Bar-Or A Calkwood JC Chognot C , et al. Effect of ocrelizumab on vaccine responses in patients with multiple sclerosis: the VELOCE study. Neurology 2020; 95: e1999–e2008. 10.1212/WNL.0000000000010380 PMC7843152 32727835

[115] Reyes S Ramsay M Ladhani S , et al. Protecting people with multiple sclerosis through vaccination. Pract Neurol 2020; 20: 435–445. 10.1136/practneurol-2020-002527 32632038

[116] Xie Y Tian Z Han F , et al. Factors associated with relapses in relapsing-remitting multiple sclerosis: a systematic review and meta-analysis. Medicine (Baltimore) 2020; 99: e20885. 10.1097/MD.0000000000020885 PMC7337585 32629678

[117] Krbot Skorić M Rogić D Lapić I , et al. Humoral immune response to COVID-19 vaccines in people with secondary progressive multiple sclerosis treated with siponimod. Mult Scler Relat Disord 2022; 57: 103435. 34920248 10.1016/j.msard.2021.103435 PMC8629510

[118] Farez MF Correale J Armstrong MJ , et al. Practice guideline update summary: vaccine-preventable infections and immunization in multiple sclerosis: report of the Guideline Development, Dissemination, and Implementation Subcommittee of the American Academy of Neurology. Neurology 2019; 93: 584–594. 31462584 10.1212/WNL.0000000000008157

[119] Otero-Romero S Lebrun-Frénay C Reyes S , et al. ECTRIMS/EAN consensus on vaccination in people with multiple sclerosis: improving immunization strategies in the era of highly active immunotherapeutic drugs. Mult Scler 2023; 29: 904–925. 37293841 10.1177/13524585231168043 PMC10338708

[120] Olberg HK Eide GE Cox RJ , et al. Antibody response to seasonal influenza vaccination in patients with multiple sclerosis receiving immunomodulatory therapy. Eur J Neurol 2018; 25: 527–534. 29205701 10.1111/ene.13537

[121] Metze C Winkelmann A Loebermann M , et al. Immunogenicity and predictors of response to a single dose trivalent seasonal influenza vaccine in multiple sclerosis patients receiving disease-modifying therapies. CNS Neurosci Ther 2019; 25: 245–254. 30044050 10.1111/cns.13034 PMC6488907

[122] Winkelmann A Metze C Frimmel S , et al. Tick-borne encephalitis vaccination in multiple sclerosis: a prospective, multicenter study. Neurol Neuroimmunol Neuroinflamm 2020; 7: e664. 10.1212/NXI.0000000000000664 PMC6984132 31919278

[123] Sabatino JJ Mittl K Rowles WM , et al. Multiple sclerosis therapies differentially affect SARS-CoV-2 vaccine-induced antibody and T cell immunity and function. JCI Insight 2022; 7: e156978. 10.1172/jci.insight.156978 PMC8876469 35030101

[124] Jalusic KO Ellenberger D Rommer P , et al. Effect of applying inclusion and exclusion criteria of phase III clinical trials to multiple sclerosis patients in routine clinical care. Mult Scler 2021; 27: 1852–1863. 33467978 10.1177/1352458520985118 PMC8521377

[125] Schweitzer F Laurent S Fink GR , et al. Age and the risks of high-efficacy disease modifying drugs in multiple sclerosis. Curr Opin Neurol 2019; 32: 305–312. 30985373 10.1097/WCO.0000000000000701

[126] Marrie RA Yu N Blanchard J , et al. The rising prevalence and changing age distribution of multiple sclerosis in Manitoba. Neurology 2010; 74: 465–471. 20071664 10.1212/WNL.0b013e3181cf6ec0

[127] Solaro C Ponzio M Moran E , et al. The changing face of multiple sclerosis: Prevalence and incidence in an aging population. Mult Scler 2015; 21: 1244–1250. 25583850 10.1177/1352458514561904

[128] Wallin MT Culpepper WJ Campbell JD , et al. The prevalence of MS in the United States: A population-based estimate using health claims data. Neurology 2019; 92: e1029–e1040. 10.1212/WNL.0000000000007035 PMC6442006 30770430

[129] Tremlett H Zhao Y Joseph J , et al. Relapses in multiple sclerosis are age- and time-dependent. J Neurol Neurosurg Psychiatry 2008; 79: 1368–1374. 18535026 10.1136/jnnp.2008.145805

[130] Coerver E Janssens S Ahmed A , et al. Association between age and inflammatory disease activity on magnetic resonance imaging in relapse onset multiple sclerosis during long-term follow-up. Eur J Neurol 2023; 30: 2385–2392. 37170817 10.1111/ene.15862

[131] Strijbis EM Coerver E Mostert J , et al. Association of age and inflammatory disease activity in the pivotal natalizumab clinical trials in relapsing-remitting multiple sclerosis. J Neurol Neurosurg Psychiatry 2023; 94(10): 792–799. 37173129 10.1136/jnnp-2022-330887

[132] Vollmer BL Wolf AB Sillau S , et al. Evolution of disease modifying therapy benefits and risks: an argument for de-escalation as a treatment paradigm for patients with multiple sclerosis. Front Neurol 2021; 12: 799138. 35145470 10.3389/fneur.2021.799138 PMC8821102

[133] Marrie RA Elliott L Marriott J , et al. Comorbidity increases the risk of hospitalizations in multiple sclerosis. Neurology 2015; 84: 350–358. 25540309 10.1212/WNL.0000000000001187 PMC4336005

[134] Zhang T Tremlett H Zhu F , et al. Effects of physical comorbidities on disability progression in multiple sclerosis. Neurology 2018; 90: e419–e427. 10.1212/WNL.0000000000004885 PMC5791796 29298855

[135] Weideman AM Tapia-Maltos MA Johnson K , et al. Meta-analysis of the age-dependent efficacy of multiple sclerosis treatments. Front Neurol 2017; 8: 577. 29176956 10.3389/fneur.2017.00577 PMC5686062

[136] McGinley MP Cola PA Fox RJ , et al. Perspectives of individuals with multiple sclerosis on discontinuation of disease-modifying therapies. Mult Scler 2020; 26: 1581–1589. 31368401 10.1177/1352458519867314

[137] Corboy JR Fox RJ Kister I , et al. Risk of new disease activity in patients with multiple sclerosis who continue or discontinue disease-modifying therapies (DISCOMS): a multicentre, randomised, single-blind, phase 4, non-inferiority trial. Lancet Neurol 2023; 22: 568–577. 37353277 10.1016/S1474-4422(23)00154-0

[138] Coerver E Fung WH de Beukelaar J , et al Discontinuation of first-line disease-modifying therapy in stable multiple sclerosis (DOT-MS): an early-terminated multicenter randomized controlled trial. Abstract Number: 1281/O099. ECTRIMS-ACTRIMS. Milan, https://s3.eu-central-1.amazonaws.com/m-anage.com.storage.congrex/abstracts_ectrims2023/146784.pdf (2023, accessed 29 July 2025).

[139] Manzano A Eskyté I Ford HL , et al. Patient perspective on decisions to switch disease-modifying treatments in relapsing-remitting multiple sclerosis. Mult Scler Relat Disord 2020; 46: 102507. 32979733 10.1016/j.msard.2020.102507

[140] Marrie RA Cohen J Stuve O , et al. A systematic review of the incidence and prevalence of comorbidity in multiple sclerosis: overview. Mult Scler 2015; 21: 263–281. 25623244 10.1177/1352458514564491 PMC4361468

[141] Marrie RA Reider N Cohen J , et al. A systematic review of the incidence and prevalence of autoimmune disease in multiple sclerosis. Mult Scler 2015; 21: 282–293. 25533299 10.1177/1352458514564490 PMC4429166

[142] Zhang T Tremlett H Leung S , et al. Examining the effects of comorbidities on disease-modifying therapy use in multiple sclerosis. Neurology 2016; 86: 1287–1295. 26944268 10.1212/WNL.0000000000002543 PMC4826339

[143] Fanouriakis A Mastorodemos V Pamfil C , et al. Coexistence of systemic lupus erythematosus and multiple sclerosis: prevalence, clinical characteristics, and natural history. Semin Arthritis Rheum 2014; 43: 751–758. 24332007 10.1016/j.semarthrit.2013.11.007

[144] Velazquez-Villoria D Macia-Badia C Segura-García A , et al. Efficacy of immunomodulatory therapy with interferon-β or glatiramer acetate on multiple sclerosis-associated uveitis. Arch Soc Esp Oftalmol 2017; 92: 273–279. 28188020 10.1016/j.oftal.2016.11.018

[145] Frahm N Hecker M Zettl UK. Polypharmacy among patients with multiple sclerosis: a qualitative systematic review. Expert Opin Drug Saf 2020; 19: 139–145. 31965869 10.1080/14740338.2020.1720646

[146] Chertcoff A Ng HS Zhu F , et al. Polypharmacy and multiple sclerosis: a population-based study. Mult Scler 2023; 29: 107–118. 36301629 10.1177/13524585221122207 PMC9896267

[147] Bachmann P Frahm N Debus JL , et al. Prevalence and severity of potential drug-drug interactions in patients with multiple sclerosis with and without polypharmacy. Pharmaceutics 2022; 14: 592. 35335968 10.3390/pharmaceutics14030592 PMC8949310

[148] Cameron MH Karstens L Hoang P , et al. Medications are associated with falls in people with multiple sclerosis: a prospective cohort study. Int J MS Care 2015; 17: 207–214. 26472941 10.7224/1537-2073.2014-076 PMC4599357

[149] Thelen JM Lynch SG Bruce AS , et al. Polypharmacy in multiple sclerosis: relationship with fatigue, perceived cognition, and objective cognitive performance. J Psychosom Res 2014; 76: 400–404. 24745782 10.1016/j.jpsychores.2014.02.013

[150] Hecker M Frahm N Bachmann P , et al. Screening for severe drug-drug interactions in patients with multiple sclerosis: a comparison of three drug interaction databases. Front Pharmacol 2022; 13: 946351. 36034780 10.3389/fphar.2022.946351 PMC9416235

[151] Confavreux C Hutchinson M Hours MM , et al. Rate of pregnancy-related relapse in multiple sclerosis. Pregnancy in Multiple Sclerosis Group. N Engl J Med 1998; 339: 285–291. 9682040 10.1056/NEJM199807303390501

[152] Alroughani R Alowayesh MS Ahmed SF , et al. Relapse occurrence in women with multiple sclerosis during pregnancy in the new treatment era. Neurology 2018; 90: e840–e846. 10.1212/WNL.0000000000005065 29429970

[153] Dobson R Jokubaitis VG Giovannoni G. Change in pregnancy-associated multiple sclerosis relapse rates over time: a meta-analysis. Mult Scler Relat Disord 2020; 44: 102241. 32521483 10.1016/j.msard.2020.102241

[154] Hellwig K Verdun di Cantogno E Sabidó M. A systematic review of relapse rates during pregnancy and postpartum in patients with relapsing multiple sclerosis. Ther Adv Neurol Disord 2021; 14: 17562864211051012. 10.1177/17562864211051012 PMC8645312 34876925

[155] Lorefice L Fronza M Fenu G , et al. Effects of pregnancy and breastfeeding on clinical outcomes and MRI measurements of women with multiple sclerosis: an exploratory real-world cohort study. Neurol Ther 2022; 11: 39–49. 34714518 10.1007/s40120-021-00297-6 PMC8857366

[156] Schubert C Steinberg L Peper J , et al. Postpartum relapse risk in multiple sclerosis: a systematic review and meta-analysis. J Neurol Neurosurg Psychiatry 2023; 94: 718–725. 36807056 10.1136/jnnp-2022-330533

[157] Krysko KM Dobson R Alroughani R , et al. Family planning considerations in people with multiple sclerosis. Lancet Neurol 2023; 22: 350–366. 36931808 10.1016/S1474-4422(22)00426-4

[158] Sandberg-Wollheim M Neudorfer O Grinspan A , et al. Pregnancy outcomes from the branded glatiramer acetate pregnancy database. Int J MS Care 2018; 20: 9–14. 29507538 10.7224/1537-2073.2016-079 PMC5825987

[159] Kaplan S Zeygarnik M Stern T. Pregnancy, fetal, and infant outcomes following maternal exposure to glatiramer acetate during pregnancy and breastfeeding. Drug Saf 2022; 45: 345–357. 35297004 10.1007/s40264-022-01168-1

[160] Lopez-Leon S Geissbühler Y Sabidó M , et al. A systematic review and meta-analyses of pregnancy and fetal outcomes in women with multiple sclerosis: a contribution from the IMI2 ConcePTION project. J Neurol 2020; 267: 2721–2731. 32444984 10.1007/s00415-020-09913-1 PMC7419441

[161] Thiel S Ciplea AI Gold R , et al. The German Multiple Sclerosis and Pregnancy Registry: rationale, objective, design, and first results. Ther Adv Neurol Disord 2021; 14: 17562864211054956. 10.1177/17562864211054956 PMC8613898 34840606

[162] Kaplan S Zeygarnik M Stern T , et al. Pregnancy and fetal outcomes following maternal exposure to glatiramer acetate in all three trimesters of pregnancy. Eur J Neurol 2023; 30(12): 3890–3895. 37565380 10.1111/ene.16036

[163] Krysko KM Rutatangwa A Graves J , et al. Association between breastfeeding and postpartum multiple sclerosis relapses: a systematic review and meta-analysis. JAMA Neurol 2020; 77: 327–338. 31816024 10.1001/jamaneurol.2019.4173 PMC6902174

[164] Ciplea AI Langer-Gould A Stahl A , et al. Safety of potential breast milk exposure to IFN-β or glatiramer acetate: one-year infant outcomes. Neurol Neuroimmunol Neuroinflamm 2020; 7: e757. 10.1212/NXI.0000000000000757 PMC7251509 32434802

[165] Ciplea AI Kurzeja A Thiel S , et al. Eighteen-month safety analysis of offspring breastfed by mothers receiving glatiramer acetate therapy for relapsing multiple sclerosis—COBRA study. Mult Scler 2022; 28: 1641–1650. 35362346 10.1177/13524585221083982 PMC9315183

[166] Ciplea AI Kurzeja A Thiel S , et al. Safety evaluations of offspring breastfed by mothers receiving glatiramer acetate for relapsing multiple sclerosis. Mult Scler Relat Disord 2023; 75: 104771. 37245349 10.1016/j.msard.2023.104771

[167] Kingwell E Zhu F Evans C , et al. Causes that contribute to the excess mortality risk in multiple sclerosis: a population-based study. Neuroepidemiology 2020; 54: 131–139. 31852000 10.1159/000504804

[168] Harding K Zhu F Alotaibi M , et al. Multiple cause of death analysis in multiple sclerosis: a population-based study. Neurology 2020; 94: e820–e829. 10.1212/WNL.0000000000008907 PMC7136054 31932517

[169] Persson R Lee S Ulcickasv Yood M , et al. Infections in patients diagnosed with multiple sclerosis: a multi-database study. Mult Scler Relat Disord 2020; 41: 101982. 32070858 10.1016/j.msard.2020.101982

[170] Knapp R Hardtstock F Krieger J , et al. Serious infections in patients with relapsing and progressive forms of multiple sclerosis: a German claims data study. Mult Scler Relat Disord 2022; 68: 104245. 36306609 10.1016/j.msard.2022.104245

[171] Medeiros Junior WLG de Demore CC Mazaro LP , et al. Urinary tract infection in patients with multiple sclerosis: an overview. Mult Scler Relat Disord 2020; 46: 102462. 32890816 10.1016/j.msard.2020.102462

[172] Lechner-Scott J Waubant E Levy M , et al. Is multiple sclerosis a risk factor for infections? Mult Scler Relat Disord 2020; 41: 102184. 32571610 10.1016/j.msard.2020.102184 PMC7204651

[173] Richter D Faissner S Bartig D , et al. Multiple sclerosis is not associated with an increased risk for severe COVID-19: a nationwide retrospective cross-sectional study from Germany. Neurol Res Pract 2021; 3: 42. 34399858 10.1186/s42466-021-00143-y PMC8364944

[174] Reder AT Centonze D Naylor ML , et al. COVID-19 in patients with multiple sclerosis: associations with disease-modifying therapies. CNS Drugs 2021; 35: 317–330. 33743151 10.1007/s40263-021-00804-1 PMC7980129

